# Prediction of Mechanical Properties and Stress–Strain Relation of Closed-Cell Aluminium Foam Under Compression Using Neural Network Models

**DOI:** 10.3390/ma18194492

**Published:** 2025-09-26

**Authors:** Anna M. Stręk, Marek Dudzik, Tomasz Machniewicz

**Affiliations:** 1 Cracow University of Technology, Faculty of Civil Engineering, ul. Warszawska 24, 31-155 Kraków, Poland; 2 Cracow University of Technology, Faculty of Electrical and Computer Engineering, ul. Warszawska 24, 31-155 Kraków, Poland; marek.dudzik@pk.edu.pl; 3 AGH University of Krakow, Faculty of Mechanical Engineering and Robotics, al. A. Mickiewicza 30, 30-059 Krakow, Poland; machniew@agh.edu.pl

**Keywords:** aluminium foam, artificial neural network, compressive stress–strain relation, plateau stress, quasi-elastic gradient

## Abstract

The presented research aims to find a data-driven formula for the compressive stress–strain behaviour of closed-cell aluminium foams with respect to the apparent density of the material. This is a continuation and new development of an earlier study by the authors. In the previous step, 500 artificial neural network models were built and trained on experimental results from compression tests and then evaluated based on, among other factors, mean absolute relative errors for training and verification stages. In this step, the evaluation of networks is amended, and criteria are introduced that are connected with the mechanical characteristics of the material, i.e., the plateau stress and quasi-elastic gradient. A weighted condition of all measures is proposed. Based on the amended conditions, a neural network model with a weighted mean absolute relative error of ≅5% is chosen and presented, together with the mathematical equation for its stress–strain–density relationship $\sigma =f\left(\varepsilon ,\rho \right)$ over a range of material apparent densities $\rho \in \left\langle 0.2;0.3\right\rangle \;g/{cm}^{3}$. Experimental relationships for compressive strength and plateau stress are also presented.

## 1. Introduction

### 1.1. Literature Overview

Aluminium foams are valued engineering materials. Much has already been written about their production, properties, and applications [1,2,3,4,5,6]. The most appreciated features of these materials include their lightweight nature and resulting favourable mechanical strength-to-weight ratio, high energy absorption, large specific surface, thermal insulation, and acoustic insulation. Their application areas range widely: civil engineering and architecture [7,8,9,10], the automotive industry [11,12,13], the aerospace industry [14,15,16], biomedical engineering [17,18,19,20], and the energy storage industry [21,22,23,24], among others.

In order to effectively use metal foams, researchers have been studying them with various methods to find the laws and relationships governing their behaviour. The first approach is, of course, traditional mechanical testing, which involves subjecting the material to characteristic loading [25,26]. These tests include uniaxial compression [27,28,29,30], uniaxial tension (due to the lack of a dedicated standard [31], documents for general metallic materials [32,33] and sandwich panels [34,35] can be used), and shear (there is no dedicated standard [31]; hence, again, a document for sandwich panels can be applied [36]). There are also works that report twisting and bending experiments [37,38,39]. In addition to mechanical testing, other traditional experimental techniques are used to understand and then model the behaviour of foams, like microscopic imaging [40], X-ray tomography [41,42], and DIC [43,44]. Classic numerical modelling uses the finite element method [45,46,47,48,49,50,51].

In recent years, however, along with traditionally utilised research techniques, artificial intelligence methods have been introduced to model the behaviour of metallic engineering materials [52,53], especially metal foams and sponges. These new approaches are promising complements or alternatives to traditional methods since they have the capacity to deal with non-linear problems, offer space for solving high-dimensional data sets, and are capable of discovering hidden patterns [54,55,56]. There is a wide variety of AI methods used in modelling [57,58], among which, the following techniques of machine learning are applied in modelling the behaviour of metal foams and sponges: artificial neural networks (ANNs, NNs) [59,60], convolutional neural networks (CNNs) [61,62], decision trees (DTs) [59,63], random forests (RFs) [64], linear regression (LR) [60], polynomial regression (PR) [60], and variational autoencoders (VAEs) [65,66]; this might not exhaust the list.

Relevant works will now be briefly discussed. One of the first applications of machine learning for researching porous metals was reported by Raj and Daniel [67]. The authors utilised NNs for the simulation of selected properties of a closed-cell aluminium foam subjected to compression: plateau stress, Young’s modulus, and energy absorption capacity. The input variables were relative density, cell anisotropy ratio, and average pore diameter. In the study, multilayer feedforward NNs were applied.

The authors of the present work also contributed to the application of neural networks in the modelling of porous metals. The research reported in one article [68] was aimed at a preliminary study on the use of neural networks to model the stress–strain relationship of an open-cell aluminium sponge subjected to compression. This approach was then developed and reported in [69]. The paper presents a methodology for building and evaluating different network architectures with the purpose of finding the optimal solution. The following evaluation criteria are given and discussed in detail: mean average relative error, coefficient of determination, root mean square error, and mean square error. Feedforward NNs with 1–50 neurons in one hidden layer were tested. As a result of the study, the authors obtained a seven-neuron model for stress–strain relations in the compression of open-cell aluminium [70]. This model, however, has to take into account the prototype character of metal sponge specimens, which were the source of the experimental compression data. Another work of the authors included studying the possibility of using neural networks to model the behaviour of closed-cell foams [71]. Aspects such as accuracy, robustness, and overfitting were considered. The results represent the first step of the present research, and hence, some of the results will be recalled in Section 1.2.

A number of articles are devoted to using machine learning for X-ray tomography image processing with the aim of retrieving porous metal features. Hangai et al. [72] reported using logistic regression (LR) for the classification of foam specimens to a group with either high compression strength or low compression strength based on its cross-sectional X-ray photographs. This classification is based on the relationship of the foam’s porosity to its compressive strength. Basically the same research team, Hangai et al., in their following works [73,74,75], focused on utilising two-dimensional CT images as data for machine learning-based prediction of plateau stress and porosity [75]. The usage of X-ray images allowed the assessment of plateau stress, because the latter is connected to porosity. The authors used convolutional neural networks, which belong to supervised deep learning methods. The importance of specimen number, image quality, and orientation was proven. The research showed that non-destructive testing can help to evaluate the compressive properties of closed-cell metal foam. Computed tomography was also the data basis for research reported by Kammbach et al. [76]. The research aimed to analyse the morphological properties of metal foams. The authors used CNN and trained it both on simulated Voronoi foams as well as radiograms of actual specimens. The training was aimed at the regression of the cell size distribution and the sphericity distribution obtained from images.

Convolutional neural networks were also adopted by Zhuang et al. for research [77]. In their study, numerical geometrical models of aluminium foam were built and voxelised and their mechanical properties were calculated by the finite element method, including the stress–strain response, densification strain, and plateau stress. Next, a deep learning data set consisting of the foam voxel model and mechanical properties was created. A three-dimensional convolutional neural network was trained to identify the mesostructure features of a foam model and the relationship between the mesostructure and the macroscale mechanical properties.

One special class of porous metallic materials is nanoporous metals. Artificial intelligence is also used in this field for modelling. In [78], Dyckhoff and Huber reported how ML was applied for modelling the multiaxial yield behaviour of nanoporous metals. The researchers first studied the elastic behaviour and yield surfaces of RVEs with idealised diamond and Kelvin structures in FEM simulations. Then, they used data-driven and hybrid ANNs, as well as data-driven support vector machines, for the prediction of yield surfaces. The results for all ML methods were compared and support vector machines turned out to provide the most favourable results.

The internal structure of nanoporous metals is fairly complex, and its adequate design and description is crucial for applications. With this aim, Romero et al. analysed microscopic images of copper–nickel nanofoams with generative AI [79]. They introduced a deep autoencoder representation that encoded microstructure patterns, as observed in microscopic images. They used a variational autoencoder (VAE), which was capable of describing hidden geometrical features derived from embedding vectors learnt during the image reconstruction task. The embedding vectors enabled the classification of Cu–Ni nanofoams.

Since metal foams may be applied as filters or heat exchangers, fluid and heat flow through these porous media have been studied, and some recent works have also included ML. However, because such research is connected more to fluid mechanics than material or structural mechanics, which is the focus of the present paper, there will now be only a few examples briefly presented for illustrative purposes. In [80], Avalos-Gauna et al. investigated the reliability of different AI methods to predict the permeability and Forchheimer coefficient of a lost carbonate sintering open-cell porous metal. The comparison involved multiple linear regression, polynomial regression, a random forest regressor, and artificial neural network. Features chosen as predictors included the porosity, pore size, and coordination number. Baiocco et al. applied two neural networks with a resilient backpropagation algorithm for the prediction of metal foam heat exchanger behaviour and to evaluate its performance in terms of the heat exchange coefficient and efficiency [81]. In [82], Calati et al. reported gathering a large database from the open literature, which was then used to train an ANN model to estimate the heat transfer performance of metal foams during water pool boiling. It was stated that the developed neural network tool could be successfully implemented to predict the intrinsic complexity of water pool boiling inside metal foams.

In the end, it is worth noting that metal foams and sponges are not the only porous materials for which AI is helpful. Since in some cases, regardless of the skeletal material, porous solids share chosen features, a few instances from related fields will now be listed as potential inspiration. Researchers apply ML, among others, for polymer cellular solids [83,84,85], polymeric lattice structures [86], ceramic cellular materials [87], or foam concrete [88,89]. Finally, one approach has also been reported which does not focus on skeletal material, but on the cellular structure itself [90].

As can be seen, recent years have brought some interesting ideas in the application of machine learning to porous metals science and have proven the potential of this research direction. This article aims to add new aspects to the field’s development. As has been remarked above, the present paper reports research which is a continuation and development of earlier studies of the authors [71]. Now, a brief summary of what was conducted and what is needed for the present step of the investigations will be given. The specific purposes and contributions of the present study will be discussed in Section 1.3.

### 1.2. Previous Research Step 

**Figure 1:**
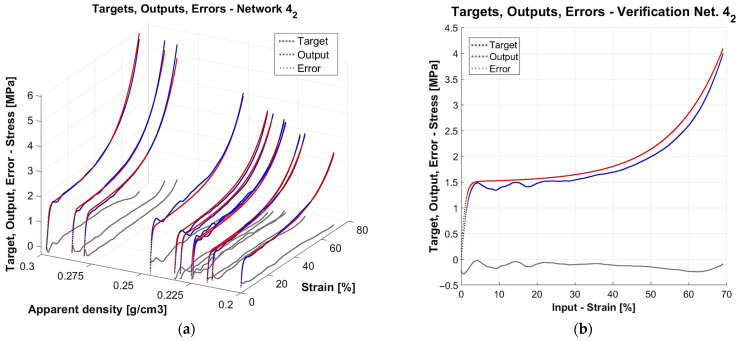
Example of results for the network with 4 neurons, Approach 2: (**a**) the model trained on data of 11 specimens, (**b**) prognosis of the model for the unknown data of the 12th specimen. Red dots represent network outputs, dark blue experimental data, and grey errors.

In the previous research stage [71], a specially designed algorithm was used to build and train 500 neural networks to model the quasi-static compressive behaviour of closed-cell aluminium foams. The models were two-layer feedforward networks with 1 to 50 neurons in the hidden layer. For each number of neurons in the hidden layers, 10 training approaches were performed. Experimental data from quasi-static compression was used for the training and verification of models. Before the main computations, the data was preprocessed (smoothed and normalised). There were altogether 12 samples subject to compression. Data from 11 out of the 12 specimens was used for the models’ supervised teaching (60% for training, 20% for validation, and 20% for test stages). The models were assessed according to assumed criteria to select the best networks. In terms of learning accuracy, the mean absolute relative error of the test stage ($MARE_{Test}$) was selected as the evaluation measure. The data of the excluded specimen was used to verify the prognosis capability of the already trained and internally validated and tested networks. In that case, the mean absolute relative error was also chosen as the evaluation measure ($MARE_{Verif}$). A detailed discussion of all the assessment criteria and obtained results is presented in [71], while here only three networks, finally chosen as the best ones, together with the values of the two above-mentioned criteria, are recalled in Table 1 for illustrative purposes. As an example, plots which depict network mapping, experimental data, and errors for one of the three networks (with four neurons, Approach 2) are shown in Figure 1.

One can observe that both mapping based on the data known from the training (Figure 1a) as well as mapping of the completely unknown data (Figure 1b), have a close resemblance to the experimental results. A question arises as to whether it would be possible to extend the domain of apparent density outside the discrete 12 values of the actual experimental specimens, and how to reliably verify the network prognosis capacity in such a case. Research attempting to resolve this issue will be shown in the present article.

### 1.3. Aims and Contributions of This Work

Mapping outside the training and verification datasets requires a new, appropriate frame of reference that would allow a reliable and as accurate as possible assessment of the prediction. Since the original input data for the network contained information about strains and densities, while the targets contained information about stresses, an idea was developed to use mechanical properties, which are indirectly hidden in the stress–strain–density data as an additional aspect for verification. Such mechanical properties depend on density, but are determined derivatively from stress–strain curves after compression tests. These are, in particular, compressive strength, plateau stress, and quasi-elastic gradient. During network training, mechanical features were not explicitly provided as input data. A neural network could, however, detect these hidden relationships during training, which would indicate the good quality of the mapping of such a particular neural model. In our article, we use exactly this idea; in addition to the common output-target error check, we also examine the agreement between material characteristics predicted by the network to the actual material characteristics hidden in the experimental stress–strain curves.

We present our research in the following sections of the article. In Section 2.1 and Section 2.2, we review the experimental results and show how they are to be used to determine characteristic mechanical properties: compressive strength, plateau stress, plateau end strain, and quasi-elastic gradient. In Section 2.3 and Section 2.4, we present the criteria for evaluating the quality of network mapping based on target-output error measures and the mechanical property mapping error. Section 2.5 presents the final weighted criterion used to select the best model. In the next section, we report and discuss the results obtained. Section 3.1 contains the results of calculating the experimental values of the chosen mechanical properties. In addition, function fitting of these values in relation to the apparent density and to each other is shown. Next, Section 3.2 shows the obtained mean absolute relative errors of individual neural models in reference to the values from the experimental dataset for the 12 samples, as well as in correspondence to the newly assumed mechanical properties. Section 3.3 outlines how all the individual and weighted criteria were fulfilled and then presents the best network. Finally, an equation describing the stress–strain–density relationship is shown.

The main contributions of the present research include:

- Finding a data-driven stress–strain model of compression of closed-cell aluminium foam for a range of apparent densities. The relationship has an engineering application potential due to its good mapping accuracy (weighted mean absolute relative error ≅5%).
- Verification of NNs’ ability to identify mechanical features associated with density (plateau stress), which was not provided directly in the training data.
- Introduction of network quality assessment criteria that were connected with mechanical properties (plateau stress and quasi-static elastic gradient), which enabled the evaluation of networks’ extrapolation capability and finding the best model.
- Finding functions correlating the experimental results of plateau stress with density and compressive strength with plateau stress.

## 2. Materials and Methods

The investigated material was closed-cell aluminium foam with a stochastic cell distribution, equivalent cell diameter 4.0 mm, and average apparent density $\rho_{av}=0.24\;g/cm^{3}$. The foam in question was Alporas foam produced by Gleich GmbH from Kaltenkirchen, Germany. The alloy composition of the skeleton material was Al + 1.5% Ca + 1.5% Ti. Computations were performed using Matlab R2022b and MS Excel 2016.

### 2.1. Experimental Compression of Closed-Cell Aluminium

**Figure 2:**
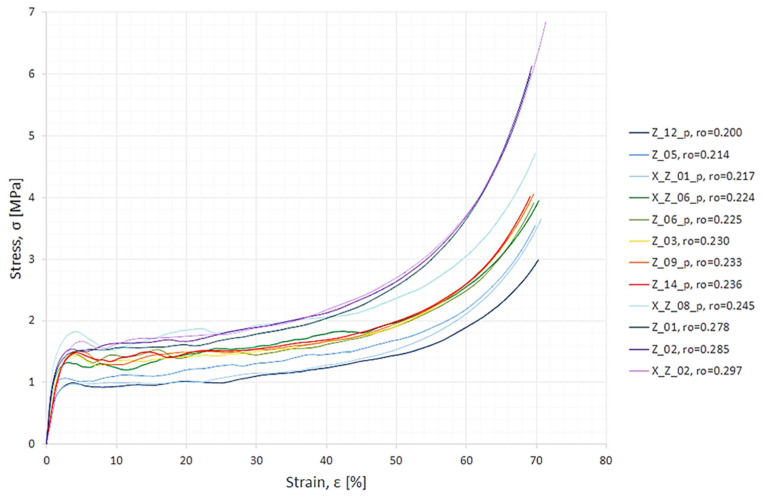
Experimental stress–strain compressive curves. After the specimen’s name is its apparent density in (g/cm^3^).

The material was cut into 5 cm × 5 cm × 5 cm cubes and quasi-statically compressed. Such specimen dimensions were assumed in order to fulfil a condition from standards [27,28]. The condition requires that the minimal sample edge length should be $\geq 10d_{0}$, where $d_{0}$ is the average pore diameter. Details of the specimens, laboratory testing, and data preprocessing can be found in [71]. Here, only the resulting stress–strain graph (Figure 2) will be recalled from [71] to serve as an illustration of the experimental data source. Additionally, from Figure 2 it can be seen that specimens had different apparent density values and that this property is linked with the given specimen’s response in the experiment. Generally, even though it is not ideally fulfilled, it can be observed that the denser the material, the higher the stress–strain curve in the diagram.

### 2.2. Determination of Mechanical Properties

**Figure 3:**
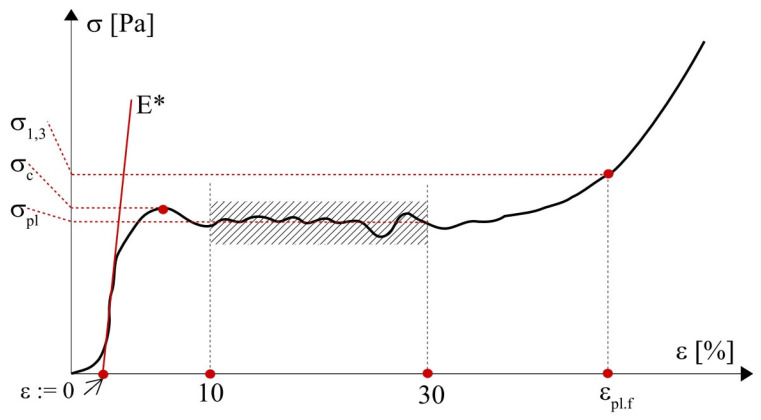
A graph representing an experimental stress–strain curve for metal foams and characteristic mechanical properties.

Procedures for determining the characteristic mechanical properties are known in the scientific literature [25,26] and are also given in standards for compression tests [27,28,29]. However, there are small differences in the definitions and methods suggested by individual literature sources. Thus, unified definitions of material characteristics and ways of their determination are given below, based on [30]. Figure 3 depicts a stress–strain curve with the following mechanical properties analysed in the present study:

- $\sigma_{c}$—compressive strength—which should be understood as the stress corresponding to the first local maximum of stresses.
- $\sigma_{pl}$—plateau stress—calculated as the arithmetic mean of plateau stresses; here assumed for stresses corresponding to strains in the range of 10–30%.
- $\sigma_{1,3}=1,3\cdot \sigma_{pl}$—auxiliary measure for calculation of plateau end.
- $\varepsilon_{pl.f}$—plateau end strain—strain corresponding to stress $\sigma_{1,3}$. Phenomenologically, it marks the beginning of the densification of the material.
- $E^{*}$—quasi-elastic gradient—understood as the initial slope of the stress–strain diagram. It should not be regarded as Young’s modulus [27,28].

In the present study, mechanical characteristics of specimens were determined based on stress–strain data from compression experiments. Inbuilt Matlab procedures for least-square fitting [91] were used to find relationships between the material’s apparent density and mechanical properties. These relations were later incorporated into calculations that combine mechanical properties and neural network modelling (see Section 2.4).

### 2.3. Neural Network Modelling and Its Quality Assessment

In the previous research step [71], 500 neural networks were generated and trained. These networks are models of the compressive behaviour of aluminium foam trained on experimental data of 11 specimens and subjected to external verification on experimental data of the 12th specimen. Mean absolute relative errors from the test stage of training (${MARE}_{Test}$) and from the verification on the extra 12th sample (${MARE}_{Verif}$) were assumed as the models’ quality measures. In the present step, the same indicators will be used.

Both measures were defined as in Relation 1 below. The criteria of satisfactory network accuracy and prognosis quality were assumed in the form of not allowing the chosen measures to exceed the assumed thresholds for the respective stages. The condition was formulated symbolically as in Relation 1:

(1) $${\left(MARE_{stage}\right)}_{n,approach}=mean\left\{{\left(\left|\frac{t_{i}-o_{i}}{t_{i}}\right|\right)}_{n,approach}\right\}\leq MARE_{stage.threshold},$$

where:

- ${MARE}_{stage}$—mean absolute relative error at the testing stage or respectively at the verification stage,
- n—given number of neurons in the hidden layer of the considered network, here n=1,2,…,50,
- approach—given number of the training repetition of the considered network, here approach=1,2,…,10,
- $t_{i}$—the i-th target for the network at the testing stage or respectively at the verification stage,
- $o_{i}$—the i-th output of the network at the testing stage or respectively at the verification stage,
- i—individual data index, should exhaust all data for the given stage,
- ${MARE}_{stage.threshold}$—the assumed threshold value for the quality condition at the respective stage.

### 2.4. Mechanical Properties from Neural Network Models

**Figure 4:**
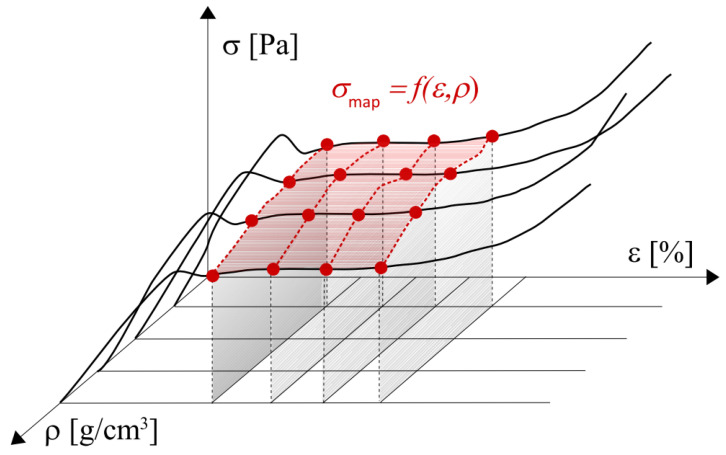
Idea of surface of stresses over the domain of strain × apparent density.

In the present research step, the NN models were used to generate stress outputs for the discrete strain range as done so earlier ($\varepsilon \in \left\langle 0;69\right\rangle \%;1000$ evenly distributed data), and apparent densities extrapolating the previous experimental data set (11 + 1 density values). Then, it was assumed that the networks should generate outputs for the discrete range of apparent densities $\rho =\left\{0.200;0.201;\ldots ;0.300\right\}\frac{g}{cm^{3}}$ (altogether 101 data items, also covering the experimental 11 + 1 values). Based on these results, graphs depicting surfaces built on mapped compressive stresses $\sigma_{map}=f\left(\varepsilon ,\rho \right)$ were plotted for each of 500 models. The idea of such a surface is shown in Figure 4 below.

The results mapped by each of the 500 models were then regarded separately for each of 101 densities as stress–strain relations (black lines in Figure 4) and mechanical properties were calculated, similarly as for the real material specimens based on their experimental compression test data. It was chosen to calculate $\sigma_{pl.map}$—plateau stress and $E_{map}^{*}$—quasi-elastic gradient. Now, having 101 mapped values for a discrete range of apparent densities for each network, it was possible to compare the results from the network with the expectation from the experiment-based fitted relations $\sigma_{pl.fit}\left(\rho \right)$ and $E_{fit}^{*}\left(\rho \right)$. Then, for each of the 500 models, 101 errors between the network prediction and the experiment-based fitted value were calculated. Next, for both mechanical properties, the mean absolute relative errors: $MARE_{\sigma_{pl}}$ and $MARE_{E^{*}}$, were calculated as well as maximum absolute relative errors: $MaxARE_{\sigma_{pl}}$ and $MaxARE_{E^{*}}$, defined as in Relations 2 and 3, respectively. Finally, the “mechanical” criteria of satisfactory network prognosis quality were postulated as not allowing the chosen measures to exceed assumed thresholds—the conditions were formulated symbolically as in Relations 2 and 3:

(2) $${\left(MARE_{prop}\right)}_{n,approach}=\mathrm{mean}\left\{{\left(\left|\frac{{prop}_{fit,j}-{prop}_{map,j}}{{prop}_{fit,j}}\right|\right)}_{n,approach}\right\}\leq MARE_{prop.threshold},$$

(3) $${\left(MaxARE_{prop}\right)}_{n,approach}=\min\left\{{\left(\left|\frac{{prop}_{fit,j}-{prop}_{map,j}}{{prop}_{fit,j}}\right|\right)}_{n,approach}\right\}\leq MaxARE_{prop.threshold},$$

where:

- ${MARE}_{prop}$—mean absolute relative error for the considered property,
- ${MaxARE}_{prop}$—maximal absolute relative error for the considered property,
- n—given number of neurons in the hidden layer of the considered network, here n=1,2,…,50,
- approach—given number of the training repetition of the considered network, here approach=1,2,…,10,
- ${prop}_{fit,j}$—property value from the experimental fitting relation for the j-th apparent density,
- ${prop}_{map,j}$—property value from the considered network mapping relation for the j-th apparent density,
- j—indicator of the given apparent density value or values, here j=1,2,…,101,
- ${MARE}_{prop.threshold}$, ${MaxARE}_{prop.threshold}$—the assumed threshold values for the quality conditions for the considered property.

### 2.5. Choice of the Best Networks

Having assumed that the number of networks complied with all individual criteria regarding prognosis ability, another condition was needed for the choice of the best of them. This meant, in other words, determining for which *n* and *approach* the best combination of the four assumed quality measure values was obtained. This condition was formulated as finding a minimum of a weighted sum $MARE_{WS.min}$, as shown in Relation 4:

(4) $$\begin{array}{c} MARE_{WS.min}=min\left\{w_{Test}\cdot {\left(MARE_{Test}\right)}_{n,approach}+w_{Verif}\cdot {\left(MARE_{Verif}\right)}_{n,approach}+\ldots \right.\\ \left.+\sum_{1}^{k}\left(w_{prop,k}{\cdot \left(MARE_{prop,k}\right)}_{n,approach}\right)\right\}, \end{array}$$

where:

- $w_{Test}$—the weight for the measure at the test stage,
- $w_{Verif}$—the weight for the measure at the verification stage,
- $w_{prop,k}$—the weight for the measure for the k-th mechanical property,
- k—number of considered mechanical properties.

Alternatively, instead of seeking the minimum value of the linear combination of measures, it would be enough for this combination to not exceed a certain value and for such networks to look for the minimum *n*. This variant of the condition is given by Relation 5:

(5) $$\begin{array}{c} w_{Test}\cdot {\left(MARE_{Test}\right)}_{n,approach}+w_{Verif}\cdot {\left(MARE_{Verif}\right)}_{n,approach}+\sum_{1}^{k}\left(w_{prop,k}{\cdot \left(MARE_{prop,k}\right)}_{n,approach}\right)\leq \ldots \\ \ldots \leq MARE_{threshold}, \end{array}$$

where:

- ${MARE}_{stage.threshold}$—the assumed threshold value for the weighted quality condition.

## 3. Results and Discussion

### 3.1. Mechanical Properties from Experiment

In accordance with the methodology described in Section 2.2, values of four characteristics of specimens used in experimental compression were determined: compressive strength $\sigma_{c.exp}$, plateau stress $\sigma_{pl.exp}$, plateau end strain $\varepsilon_{pl.f.exp}$, and quasi-elastic gradient $E_{exp}^{*}$. The results are shown in Table 2. Additionally, apparent density $\rho_{exp}$ is presented in the last column.

For each material property, a function that could represent its relation to density was sought. Attempts were also made to find if the values were interconnected. In [92], the authors suggest that for closed-cell foams the relationship between plastic yield point and density should have a quadratic form—Formula (6):

(6) $$\frac{\sigma_{pl}^{*}}{\sigma_{y}}=C_{1}{\left(\frac{\rho }{\rho_{s}}\right)}^{2}\;\Rightarrow \;\sigma_{pl}^{*}=\frac{C_{1}\sigma_{y}}{\rho_{s}^{2}}\rho^{2}\;\Rightarrow \;\sigma_{pl}^{*}\sim \rho^{2},$$

where:

- $\sigma_{pl}^{*}$—plastic yield point of foam,
- $\sigma_{y}$—plastic yield point of skeleton material,
- ρ—apparent density of foam,
- $\rho_{s}$—apparent density of skeleton material,
- $C_{1}$—constant to be determined experimentally.

**Figure 5:**
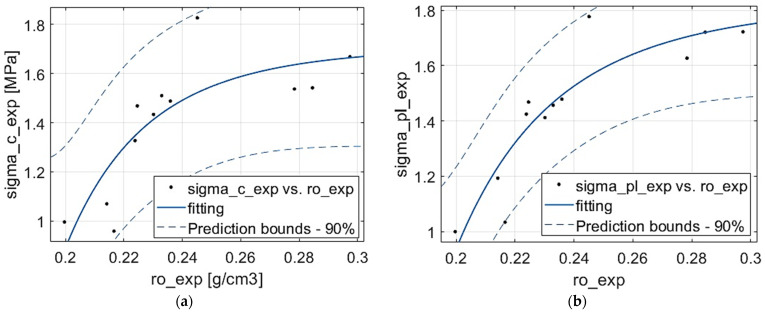
Function fitting for: (**a**) compressive strength, (**b**) plateau stress.

For the purpose of the present research, it was assumed that the plastic yield point corresponded to the compressive stress and fitting calculations were performed. They showed that the quadratic type of relationship did not model the experimental data—the coefficient of determination was very small and negative: $R^{2}=-0.010$. Hence, another relationship type was assumed, and the power function relation type was chosen to fit the compressive stress and also plateau stress. Table 3 provides the numerical results of the fitting and Figure 5 presents the graphical solutions. Judging by error measures from Table 3, the experimental results of the plateau stress reveal a more regular dependence on apparent density. However, in both properties, the correlation is satisfactory but not very good: $R_{\sigma_{c}}^{2}\approx 0.7$ and $R_{\sigma_{pl}}^{2}\approx 0.8$. This could be the effect of a few noticeable outliers (compare Figure 5), as well as the irregularity of the stochastic foam material itself.

**Figure 6:**
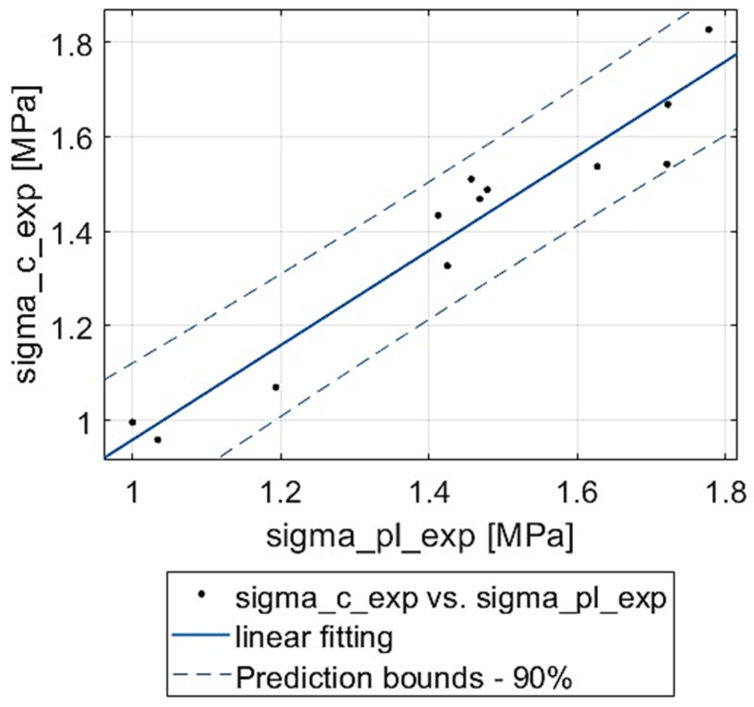
Correlation between compressive strength and plateau stress of experimental specimens.

Next, it was verified whether the experimental values of compressive stress and plateau stress are related. A linear function fitting of both measures was performed. The results are shown in Table 4 and Figure 6. It can be noticed that the properties discussed are well correlated: $R^{2}\approx 0.9$. The experimental values are a little scattered around the fit, but there are no distinct outliers. Interestingly, the fitting line is almost at 45° and with a small negative vertical shift.

**Figure 7:**
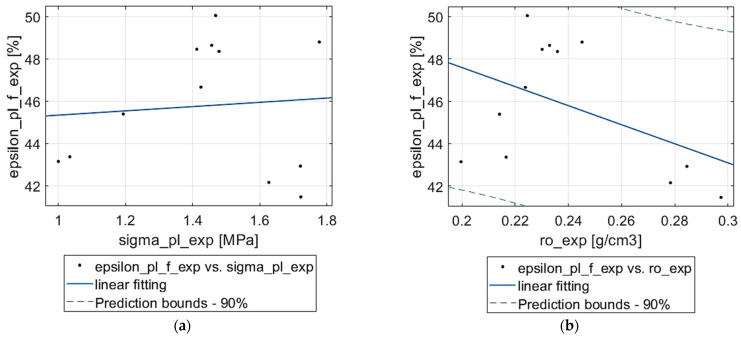
Function fitting for plateau end strain: (**a**) vs. plateau stress, (**b**) vs. apparent density.

Following this, it was checked if the experimentally obtained values of the plateau end strain show a dependence on the plateau stress and density. The results can be seen in Figure 7. Judging by the considerable scatter of the experimental points in both cases, it was decided to only verify linear relations; however, it was already visible that the results would be poor. In the case of the dependency on plateau stress, the coefficient of determination is almost zero $R_{\sigma_{pl}}^{2}=0.007$, and for density it is as low as $R_{\rho }^{2}=0.202$. This lack of relationship can be attributed again to the stochastic character of the porous material. Each specimen had a unique pore distribution and thus a different deformation path for the collapse of individual pores under compression.

Finally, the dependence of quasi-elastic gradient on density was examined. According to [92], the relation between Young’s modulus and density for closed-cell foams should have a cubic form—Formula (7):

(7) $$\frac{E}{E_{s}}=C_{2}{\left(\frac{\rho }{\rho_{s}}\right)}^{3}\Rightarrow E=\frac{C_{2}E_{s}}{\rho_{s}^{3}}\rho^{3}\Rightarrow E\rho^{3},$$

where:

- E—Young’s modulus of foam,
- $E_{s}$—Young’s modulus of skeleton material,
- ρ—apparent density of foam,
- $\rho_{s}$—apparent density of skeleton material,
- $C_{2}$—constant to be determined experimentally.

**Figure 8:**
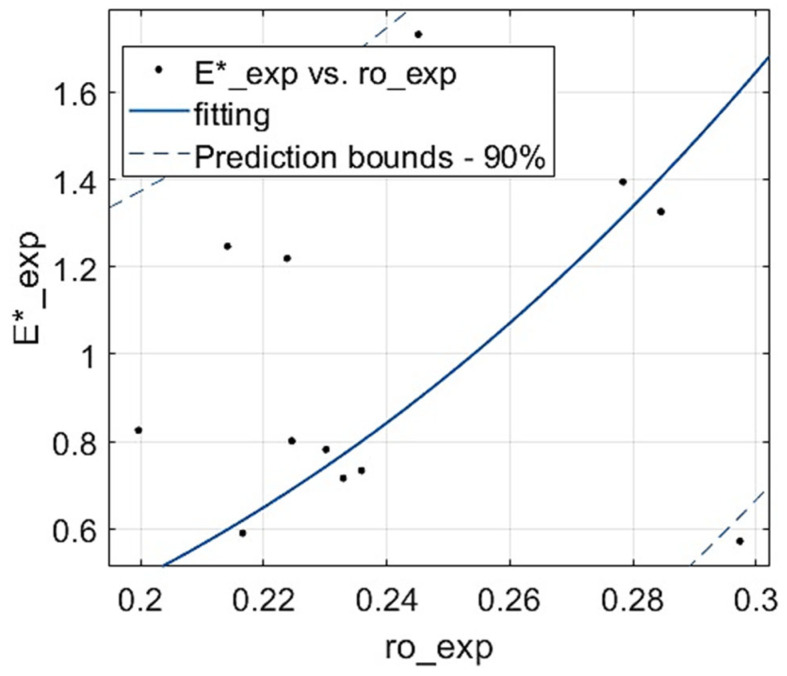
Fitting attempt for quasi-elastic gradient vs. density.

For the purpose of finding the experimental fit, it was assumed that Young’s modulus from Formula (7) would correspond to the quasi-elastic gradient. Fitting calculations proved that the cubic type of relation did not model the compression tests results and the coefficient of determination was negative: $R^{2}=-0.714$. Since experimental points were considerably scattered (Figure 8), it was assumed that no other fitting search would be performed and, for further analysis, the mean value of the experimental quasi-elastic gradients would be taken into account. Such a dispersion of experimental values is rather strange and could be attributed to the irregularity of the stochastic foam material or imperfections in the shape of the specimens.

### 3.2. Results for Mechanical Properties from Neural Network Models

**Figure 9:**
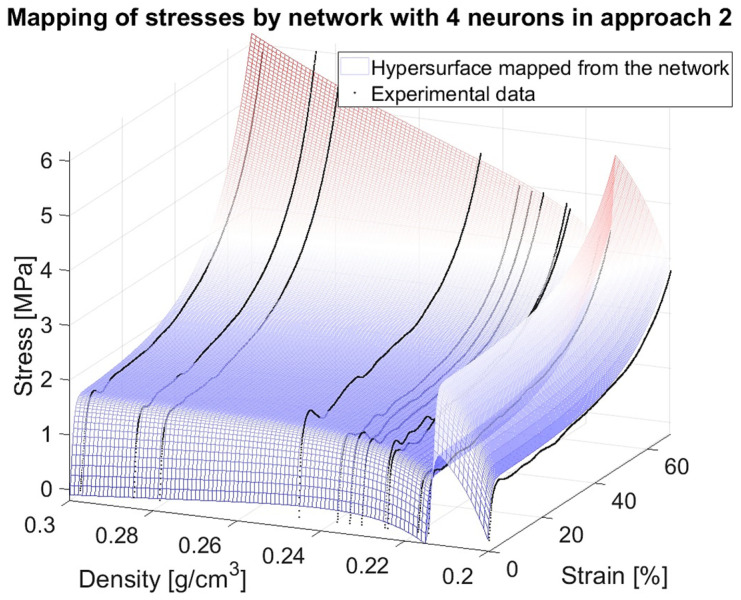
Stress surfaces for the chosen networks with 4 neurons in Approach 2.

According to the methodology described in Section 2.4, a stress surface was generated for each of 500 networks. Let us now present an exemplary surface (Figure 9) for the first of the three networks listed in Table 1. Even though the network fulfilled the criteria for $MARE_{Test}$ and $MARE_{Verif}$, it is visible that the model mapped locally inadequate surfaces when predicting outside the data for the 11 training and one verification specimens. Such a situation could have also taken place for any other of the 500 models, including the other ones with satisfactory values of mean absolute relative errors. Manual checking of so many results is, of course, not rational, so this showed the need for an auxiliary criterion or criteria that would stretch outside the directly available experimental results for 12 samples. This fact led to the introduction of new criteria connected with how well the models could predict mechanical values over an extrapolated range of densities: 101 density data items including the 12 experimental values. The general form of the criteria is given by Relation 2 in Section 2.4 and details of the realisation will be described below.

Analyses in Section 3.1 showed that the best correlation to density is reached for plateau stress and that it was possible to find the fitted formula linking the two properties (Table 2, Figure 5b). For this reason, plateau stress was chosen as the main mechanical property to be verified by the new criteria. Since compressive strength was very closely correlated with plateau stress and slightly less correlated with density than plateau stress, it was assumed that this feature would not be taken into account, because it would be enough to include only plateau stress.

On the other hand, even though it was not possible to find a fitted function for the quasi-elastic gradient, it was decided that its mean experimental value would be assumed as another measure for model verification; however, due to the lack of fitting, not as the primary but as an auxiliary measure. Additionally, for plateau end strain, no relationship to stress or density was found. It was then excluded as an indicator for the new criterion, because otherwise one would have to take into account its mean value, which would be only another approximate measure.

Having chosen the two mechanical properties (plateau stress and quasi-elastic gradient) as measures to be used in the criterion given by Relation 2, their values were calculated. Plateau stress was computed individually for each of 101 densities for all 500 models. Then, absolute relative errors in comparison to 101 values resulting from the fitted relation were determined, and finally, the mean absolute relative error $MARE_{\sigma_{pl}}$ and maximum absolute relative error $MaxARE_{\sigma_{pl}}$ for the given model were obtained. In the case of the quasi-elastic gradient, it was calculated individually for each of the 101 densities for all 500 networks, with the average then taken from the results. The average from the models was compared to the mean value from the experiments; so, in this case, instead of the mean absolute error, the absolute relative error $MARE_{E^{*}}\to ARE_{E^{*}}$ was determined and no maximum absolute relative error was sought.

**Figure 10:**
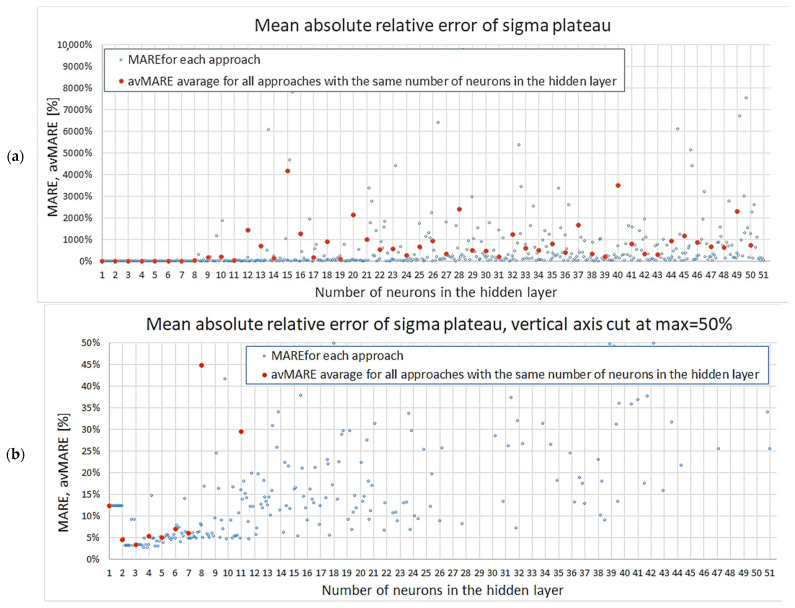
Mean absolute relative error of sigma plateau: (**a**) all results, (**b**) results up to 50%.

A summary of $MARE_{\sigma_{pl}}$ results concerning plateau stress for all networks is given in Figure 10a. The graph shows all obtained values; however, due to the very large errors of some networks, the variability of smaller results is obscured. Thus, a complementary graph with the vertical axis cut at 50% is enclosed below (Figure 10b). It can be observed from these figures that the networks with ≤7 neurons achieved little scatter of results and that for the networks with 11 neurons the scatter is considerable. This is in agreement with the conclusions of our previous study that stated that 11 neurons was the boundary from which overfitting was a risk.

**Figure 11:**
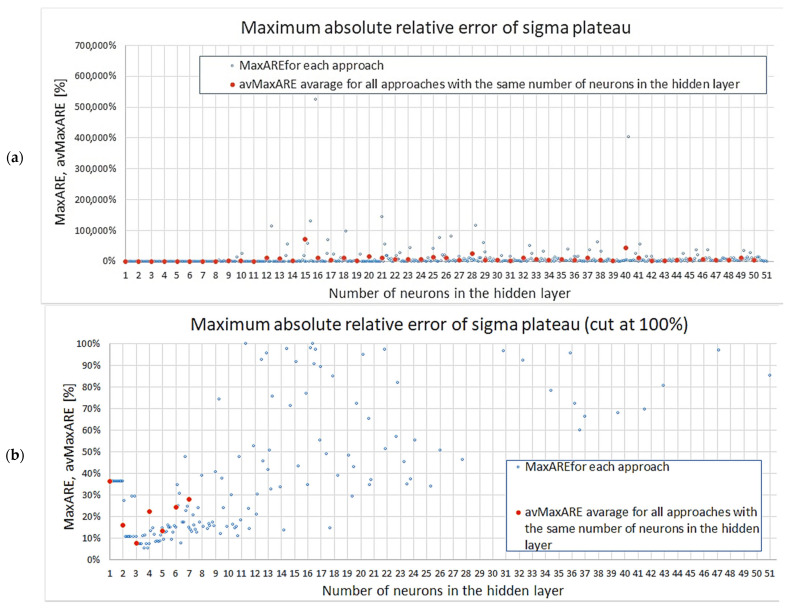
Maximum absolute relative error of sigma plateau: (**a**) all results, (**b**) results up to 100%.

Regarding the maximum absolute relative error $MaxARE_{\sigma_{pl}}$, the results are depicted in Figure 11a (all results) and Figure 11b (the vertical axis cut at 100%). The first plot due to scaling does not clearly show the variability of smaller errors, but it can be seen in the second graph. Once again, it can be noticed that the networks with seven or fewer neurons produce results with less scatter, and that all except one instance achieved $MaxARE_{\sigma_{pl}}\leq 40\%$. On the other hand, for networks with 11 neurons, almost all models, except for four cases, obtained $MaxARE_{\sigma_{pl}}\geq 30\%$.

**Figure 12:**
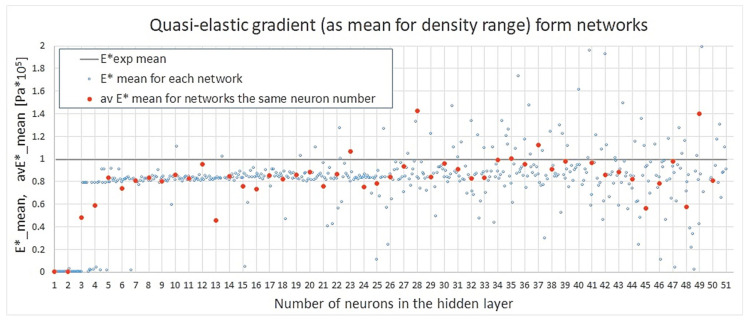
Mean quasi-elastic gradient from networks.

Figure 12 below presents the mean values of the quasi-elastic gradient obtained by all networks. It is easy to observe that the vast majority of models underestimated the magnitude of this property. This could be the effect of scattered experimental data. However, on the other hand, this indicates that the initial slopes of stress–strain curves constituted a challenge for the assumed networks in general. This means that maybe in future research, the number of hidden layers, activation functions, or input preprocessing could be calibrated so that the first linear deformation region is more accurately mapped.

**Figure 13:**
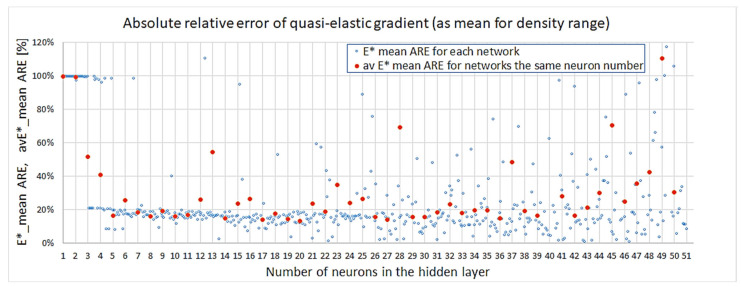
Absolute relative error for quasi-elastic gradient.

The plot in Figure 13 shows the values of the obtained absolute relative errors for all NN models. Satisfactory values start for networks with ≥4 neurons. The higher the number of neurons, the larger the error dispersion among approaches; yet, still, until reaching 21 neurons, the relative error for the mean quasi-elastic gradient stays rather consistently under 20%.

### 3.3. Choice of the Best Network

**Figure 14:**
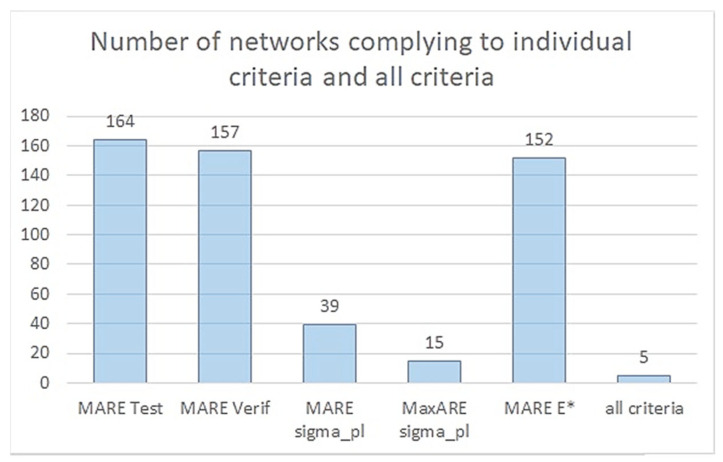
Number of networks fulfilling individual criteria.

**Figure A1:**
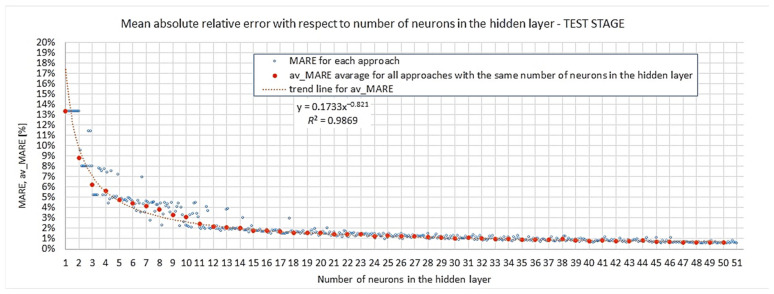
Mean absolute relative error from the test stage (from [71]).

**Figure A2:**
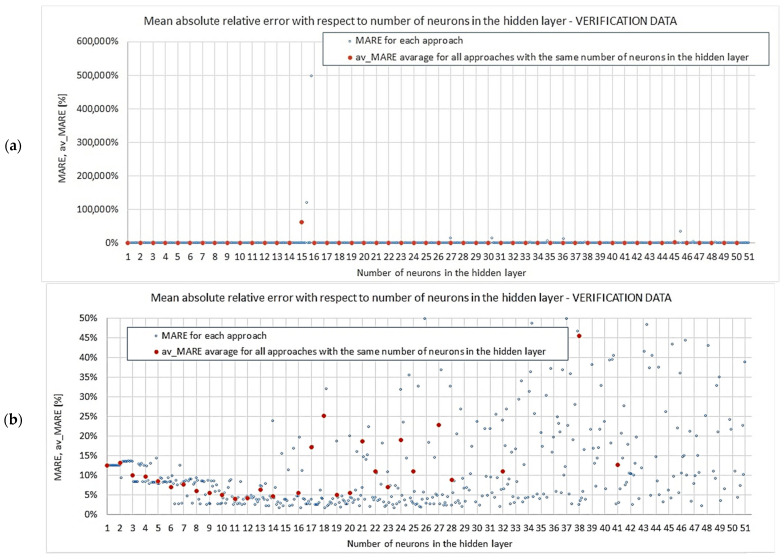
Mean absolute relative error from verification of external data: (**a**) all results, (**b**) results up to 50%. (from [71]).

Knowing the values of $MARE_{Test}$ and $MARE_{Verif}$ from the previous study step ([71], Figure A1 and Figure A2a,b in Appendix A) as well as $MARE_{\sigma_{pl}}$, $MaxARE_{\sigma_{pl}}$, and $ARE_{E^{*}}$, determined now, thresholds were chosen in order to specify the criteria conditions given in general forms by Relations 1–3. The threshold for the quasi-elastic gradient was presumed to be higher than the others, because of the experimental results being scattered and the overall underestimation trend of numerical models. Final individual criteria are shown in Relations 8–12 below. For all 500 models, individual criteria were checked. Figure 14 shows how many networks fulfilled the criteria separately as well as all of them together.

(8) $${\left(MARE_{Test}\right)}_{n,approach}\leq 5\%,$$

(9) $${\left(MARE_{Verif}\right)}_{n,approach}\leq 10\%,$$

(10) $${\left(MARE_{\sigma_{pl}}\right)}_{n,approach}\leq 5\%,$$

(11) $${\left(MaxARE_{\sigma_{pl}}\right)}_{n,approach}\leq 10\%$$

(12) $${\left(ARE_{E^{*}}\right)}_{n,approach}\leq 20\%,$$

Fulfilment of individual criteria is required, but is not enough, so a weighted condition in two alternative forms was proposed: Relation 4 for finding one best network and Relation 5 for finding a set of best networks. It was decided that only the averaged errors would be taken into account and that the maximum absolute relative error would not be included. Considering the obtained values of the four verification measures and their importance, the following weights were proposed: $w_{Test}=0.35$; $w_{Verif}=0.15$; $w_{\sigma_{pl}}=0.40$ and $w_{E^{*}}=0.10$. Let us name the weighted sum as $MARE_{Total}$ (Relation 13), and hence, the new conditions now have the form expressed by Relations 14 and 15 below. The threshold was assumed to be 5%.

(13) $$\begin{array}{c} {\left(MARE_{Total}\right)}_{n,approach}=0.35\cdot {\left(MARE_{Test}\right)}_{n,approach}+0.15\cdot {\left(MARE_{Verif}\right)}_{n,approach}+\ldots \\ \;\;\;\;\;\;\;\;\;\;\;\;\;\;\;\;\;\;\;\;\;\;\;\;\;\;\ldots +0.40\cdot {\left(MARE_{\sigma_{pl}}\right)}_{n,approach}+0.10\cdot {\left(ARE_{E^{*}}\right)}_{n,approach}, \end{array}$$

(14) $$MARE_{Total.min}=min\left\{{\left(MARE_{Total}\right)}_{n,approach}\right\},$$

(15) $${\left(MARE_{Total}\right)}_{n,approach}\leq 5\%,$$

**Figure 15:**
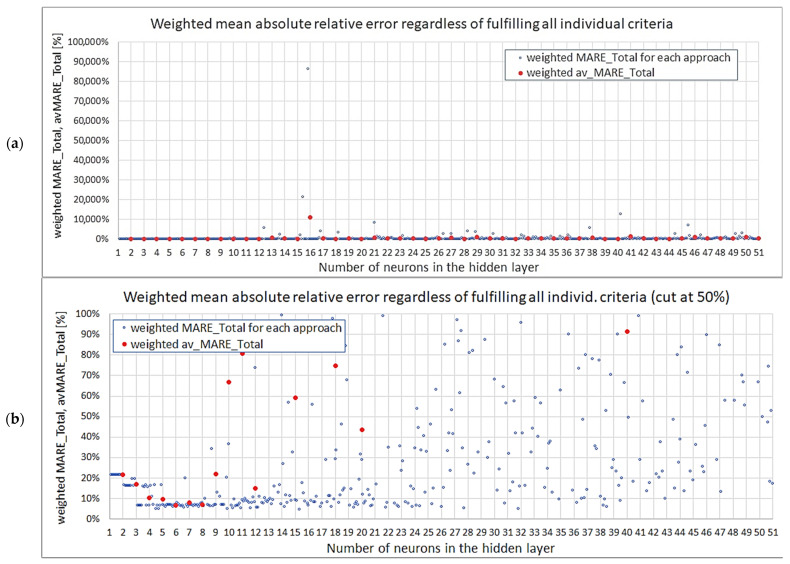
Weighted mean absolute relative error regardless of fulfilling all individual criteria: (**a**) all results, (**b**) results up to 50%.

A summary of results for the $MARE_{Total}$ for all models is presented in Figure 15a; however, due to very large values for some networks, the variability of smaller errors is obscured. Hence, a complementary plot with the vertical axis cut at 100% is given below (Figure 15b).

In Table 5, detailed results are shown for models that fulfilled all individual demands. Interestingly, none of the networks in Table 1, judged in the previous research step solely by the first two criteria, fulfil the amended conditions. This implies that adding conditions connected with the mechanical properties of the material in the extrapolated density region improved the choice via the elimination of ostensibly accurate networks. Models fulfilling all individual criteria have between four and six neurons, which confirms the conclusion from the earlier study that four is the minimal number of neurons that produces reasonable results.

**Figure 16:**
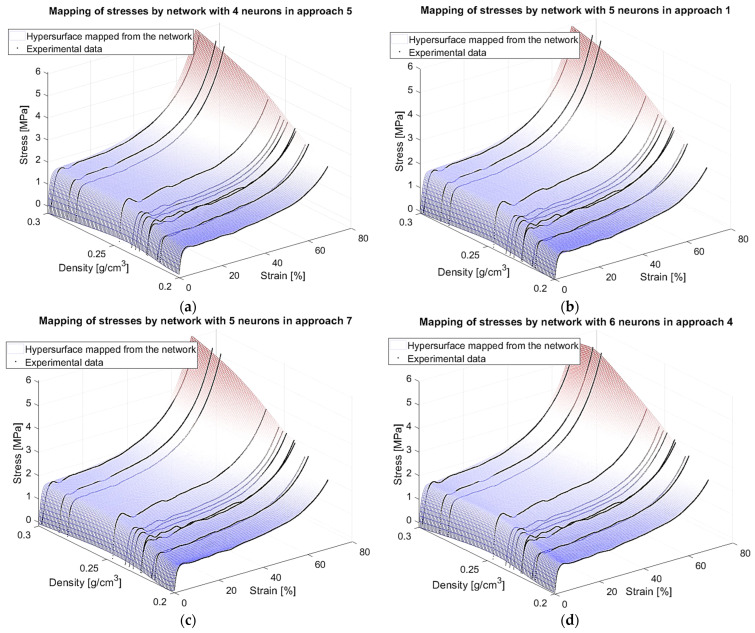
Stress surface mapping: (**a**) Model (4,5); (**b**) Model (5,1); (**c**) Model (5,7); (**d**) Model (6,4).

The minimum value of the weighted mean absolute relative error was achieved for the models with four neurons in the 5th and 7th approaches:${\left(MARE_{Total}\right)}_{4,5}={\left(MARE_{Total}\right)}_{4,7}=4.997\%\leq 5\%$. Additionally, these NNs are the only ones which fulfil the condition expressed in Relation 15 and so can be chosen as the best ones. Actually, these two networks found exactly the same model (results for other measures are also identical). This is a known situation for networks with small architectures. Formally then, we will now assume that network $\left(n,approach\right)=\left(4,5\right)$ is a representative for both and the best one found. However, it should be noted that other models in Table 5 also achieved good results and, in particular, Network (6,4) has a remarkably low $MaxARE_{\sigma_{pl}}$—the third lowest value. Figure 16 portrays how these networks map the stress surface.

**Figure 17:**
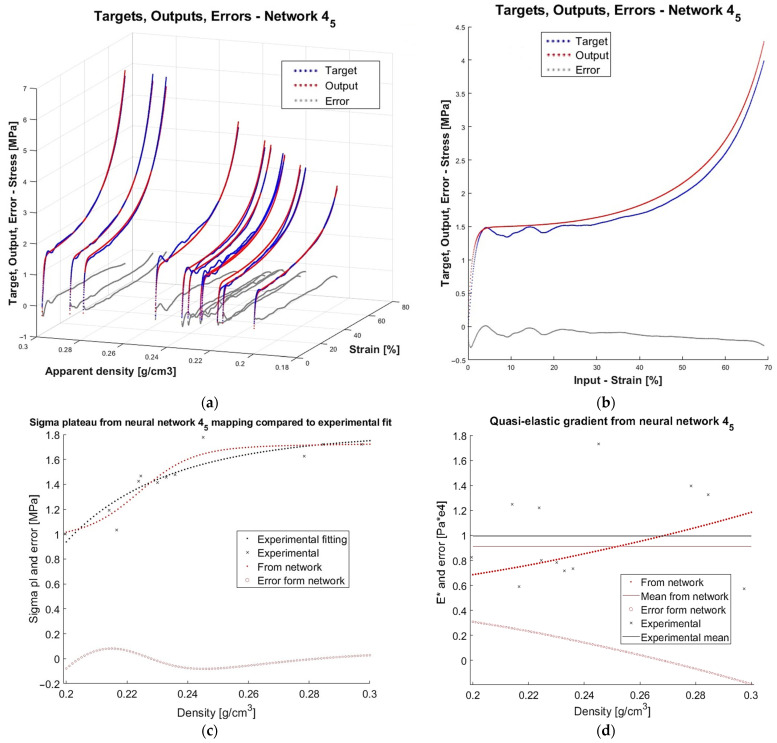
Results for the chosen best network with 4 neurons in Approach 5: (**a**) the model trained on data of 11 specimens; (**b**) verification of the model for the unknown data of the 12th specimen; (**c**) mapping of plateau stress; (**d**) mapping of quasi-elastic gradient.

**Figure A4:**
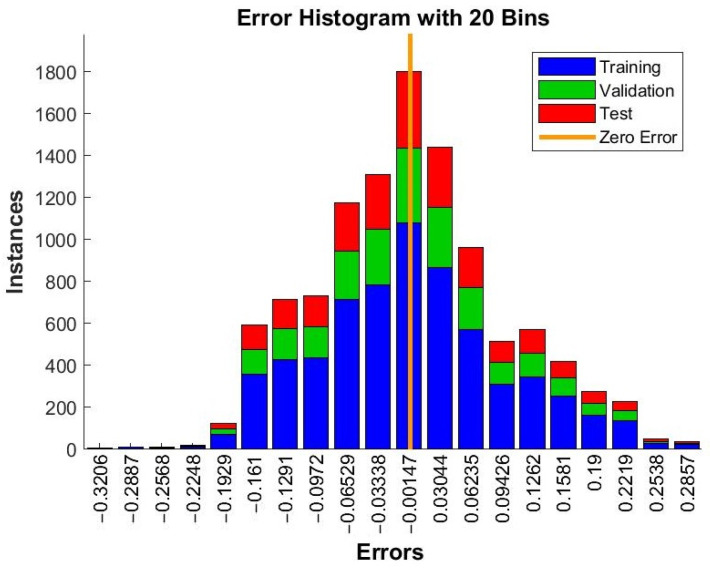
Error histogram for the chosen best network with 4 neurons in Approach 5.

**Figure A5:**
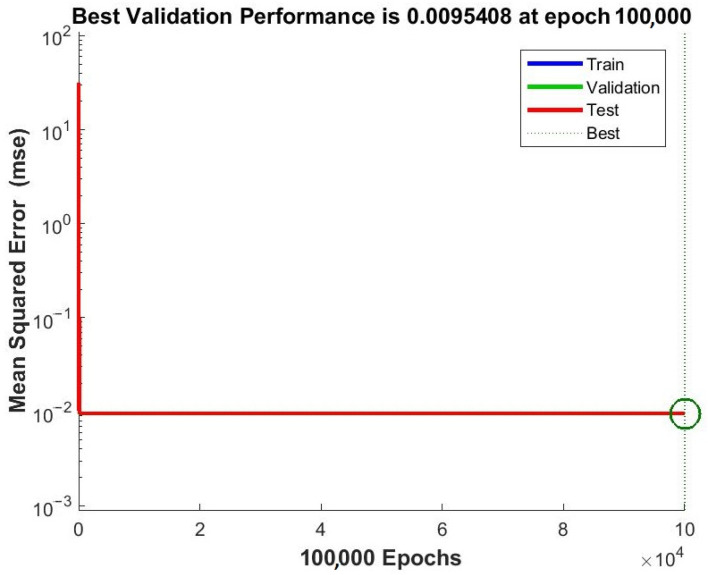
Performance for the chosen best network with 4 neurons in Approach 5.

Finally, the details and the equation of the best found model for the stress–strain–density relationship for the compression of closed-cell aluminium foam will be given. The mathematical form of the modelled relationship valid in the domain ranges $\varepsilon \in \left\langle 0;69\right\rangle \%$ and $\rho \in \left\langle 0.2;0.3\right\rangle \;g/{cm}^{3}$ is given in Equation 16, with additional explanations in 17–22. Figure 17 provides results from training, verification, plateau stress, and quasi-elastic gradient mapping. Due to the auxiliary character, diagrams presenting regression, error histogram, and training performance are shifted to Appendix B—Figure A3, Figure A4 and Figure A5. Let us note that the best network has only four neurons in one hidden layer and, at the same time, has satisfactory accuracy. The benefit of such a small architecture is its low complexity and low computation time, making the formula suitable for use in actual engineering applications. Yet, with only four neurons, the first local stress maximum corresponding to compressive strength has not been learnt by the network. This can be helped, since we provide the fitted empirical relation between the compressive strength and plateau stress in Table 4.

(16) $$\sigma ={mapminmax}^{-1}\left(W_{2}\cdot \left[{tanh}_{V}\left(W_{1}\cdot mapminmax({\left[\varepsilon \;\;\rho \right]}^{T})\right)+b_{1}\right]+b_{2}\right),$$

where

(17) $${tanh}_{V}\left({\left[V_{1}V_{2}\ldots V_{n}\right]}^{T}\right)={\left[\tanh\left(V_{1}\right)\;\;\;\tanh\left(V_{2}\right)\;\;\ldots \;\;\tanh\left(V_{n}\right)\right]}^{T},$$

(18) $$W_{1}=\left[\begin{array}{cc} 1.4289 & -0.0483\\ -1.4239 & 0.0464\\ 17.4079 & -0.1336\\ -0.3976 & 2.8045 \end{array}\right],$$

(19) $$W_{2}=[-12234.5132\;\;-36851.7386\;\;\;12053.7137\;\;\;0.1181],$$

(20) $$b_{1}={\left[-3.9650\;\;\;4.5097\;\;\;22.8168\;\;\;1.2320\right]}^{T},$$

(21) $$b_{2}=12562.9132,$$

and mapminmax—inbuilt Matlab functions [93] for normalisation and denormalisation, equivalent to:

(22) $$V^{\prime }=\frac{V-V_{min}}{V_{max}-V_{min}}\cdot \left({V^{\prime }}_{max}-{V^{\prime }}_{min}\right)+{V^{\prime }}_{min},$$

where $V^{\prime }$—the new value; V—the original value; $V_{max},V_{min}$—original interval limits; ${V^{\prime }}_{max},{V^{\prime }}_{min}$—new limits, here: −1 and 1; for output denormalisation: $V_{\sigma ,max}=5.9871\;MPa,V_{\sigma ,min}=0\;MPa$.

## 4. Conclusions

The aim of the presented research was to re-explore and develop the results obtained in the first step of the study [71] and to add new concepts in order to find a stress–strain–density relationship for aluminium foams in compression with artificial neural networks. Mean absolute relative errors from training and the verification of networks were adopted from the earlier stage. New quality indicators connected with the experimentally determined mechanical properties of the material were added to these measures: the mean and maximum absolute relative error for plateau stress and absolute relative error for the mean quasi-elastic gradient. Finally, a weighted sum of the indicating factors was used for choosing the best network.

Regarding the main contribution, with this research we have shown that in order to successfully map a stress–strain relationship for a range of apparent densities with neural networks it is beneficial not only to take traditional measures for NNs like target-output errors, but also indicators connected with the material’s mechanical characteristics that are indirectly associated with input/output datasets. Additionally, it was possible to obtain a mathematical formula for this relationship, which due to its relatively low complexity, can potentially be used in actual engineering applications. Another contribution of this paper is to provide an experiment-based function that associates two mechanical characteristics: compressive strength and plateau stress.

On the other hand, the initial region of the stress–strain curves, namely the linear slope and the first local maximum, was the most challenging for modelling (scattered values of quasi-elastic gradient and no distinct mapping of compressive strength). This implies that future research could focus on this aspect.

## Figures and Tables

**Table 1:** Networks chosen in [71] as the best ones.

Number of Neurons in the Hidden Layer	Approach	$MARE_{Test}$	$MARE_{Verif}$
4	2	4.455%	8.688%
6	6	3.572%	2.689%
11	4	1.959%	2.976%

**Table 2:** Mechanical properties of specimens used in the compression testing.

Sample Name	$\sigma_{c.exp}$ (Mpa)	$\sigma_{pl.exp}$ (Mpa)	$\varepsilon_{pl.f.exp}$ (%)	$E_{exp}^{*}$ $(10^{5}$ Pa)	$\rho_{exp}$ (g/cm^3^)
X_Z_02	1.668	1.722	41.465	0.571	0.297
Z_01	1.537	1.627	42.154	1.395	0.278
Z_02	1.542	1.721	42.924	1.326	0.285
Z_03	1.434	1.412	48.451	0.782	0.230
Z_05	1.070	1.193	45.389	1.247	0.214
X_Z_01_p	0.959	1.034	43.363	0.590	0.217
X_Z_06_p	1.327	1.425	46.655	1.220	0.224
X_Z_08_p	1.827	1.777	48.791	1.733	0.245
Z_06_p	1.469	1.468	50.042	0.801	0.225
Z_09_p	1.510	1.457	48.635	0.716	0.233
Z_12_p	0.996	1.000	43.144	0.826	0.200
Z_14_p	1.488	1.479	48.347	0.734	0.236
mean	1.403	1.437	46.005	0.931	0.240
median	1.469	1.457	46.655	0.801	0.236
standard deviation	0.240	0.230	2.807	0.392	0.027
coefficient of variation	17.12%	16.03%	6.10%	42.14%	11.32%

**Table 3:** Results of function fitting for experimental compressive strength and plateau stress in relation to apparent density.

Result of Fitting	Compressive Strength	Plateau Stress
Function formula *	$\sigma_{c.fit}\left(\rho \right)=-7.432\cdot 10^{-6}\cdot \rho^{-7.214}+1.711$	$\sigma_{pl.fit}\left(\rho \right)=-7.005\cdot 10^{-5}\cdot \rho^{-5.875}+1.833$
Coefficient of determination	$R^{2}=0.683$	$R^{2}=0.824$
Sum of Square Errors (Mpa^2^)	SSE=0.250	SSE=0.128
Root Mean Squared Error (Mpa)	RMSE=0.167	RMSE=0.120

* Stress is expressed in (Mpa) and density in ($\frac{g}{{cm}^{3}}$).

**Table 4:** Results of correlation between experimental compressive strength ($\sigma_{c.exp}$) and plateau stress ($\sigma_{pl.exp}$).

Result of Fitting	Compressive Strength
Function formula	$\sigma_{c.exp}\left(\sigma_{pl.exp}\right)=1.001\cdot \sigma_{pl.exp}-0.0417$
Coefficient of determination	$R^{2}=0.925$
Sum of Square Errors (MPa^2^)	SSE=0.0593
Root Mean Squared Error (MPa)	RMSE=0.0770

**Table 5:** Criteria measures for 17 networks fulfilling the assumed conditions.

Number of Neurons and Approach n,approach	$MARE_{Test}$	$MARE_{Verif}$	$MARE_{\sigma_{pl}}$	$MaxARE_{\sigma_{pl}}$	$ARE_{E^{*}}$	$MARE_{Total}$
4,5	4.99%	8.18%	2.98%	8.40%	8.31%	4.997%
4,7	4.99%	8.18%	2.98%	8.40%	8.31%	4.997%
5,1	4.85%	7.91%	3.93%	9.36%	19.67%	6.422%
5,7	4.97%	8.18%	4.56%	9.30%	18.87%	6.678%
6,4	4.69%	8.71%	4.10%	7.73%	16.44%	6.232%

**Figure A3:**
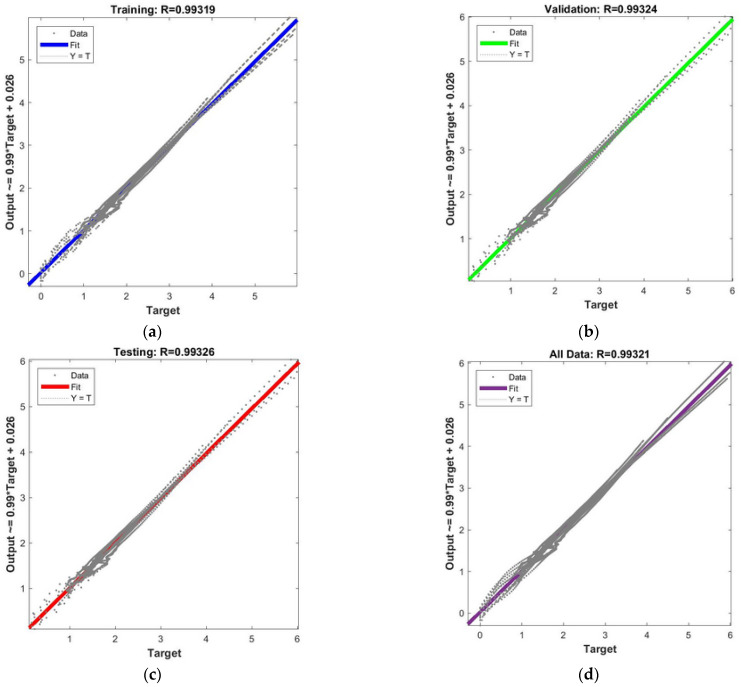
Results of regression for the chosen best network with 4 neurons in approach 5: (**a**) the trained stage; (**b**) the validation stage; (**c**) the testing stage; (**d**) all stages.

## Data Availability

The original contributions presented in this study are included in the article. Further inquiries can be directed to the corresponding author.

## Abbreviations

The following abbreviations are used in this manuscript:

AI	Artificial Intelligence
ANN(s)	Artificial Neural Network(s)
ARE	Absolute Relative Error
CNN(s)	Convolutional Neural Network(s)
CT	Computed Tomography
DIC	Digital Image Correlation
DT	Data Tree
FEM	Finite Element Method
LR	Linear Regression
LR	Logistic Regression
MARE	Mean Absolute Relative Error
ML	Machine Learning
NN(s)	Neural Network(s)
PR	Polynomial Regression
RF	Random Forest
RMSE	Root Mean Squared Error
RVE(s)	Representative Volume Element(s)
SSE	Sum of Square Errors
VAE(S)	Variational Autoencoder(s)

## Appendix A

Below are figures presenting mean absolute relative errors from the test stage (Figure A1) and for verification data (Figure A2a,b). The last graph was cut to 50% at the vertical axis for the clarity of presenting data. All plots are cited from the article presenting the previous step of the research [71].

## Appendix B

Figure A3, Figure A4 and Figure A5 below present network training results for the chosen best model with four neurons in approach 5.
